# Challenges and career consequences of internationally educated nurses: Empirical research qualitative

**DOI:** 10.1002/nop2.1977

**Published:** 2023-08-21

**Authors:** Chiharu Miyata

**Affiliations:** ^1^ Department of Nursing Sciences Mie University Graduate School of Medicine Health Sciences Tsu Japan

**Keywords:** career support, internationally educated nurses, qualitative content analysis

## Abstract

**Aim:**

To explore challenges, and career consequences of internationally educated nurses, and considerations of development policy, education, and support for them from the perspective of nursing administrators.

**Design:**

This study applied a qualitative description design.

**Methods:**

A semi‐structured interview guideline were used, interviewed eight internationally educated nurses and nine nursing administrators were chosen based on a purposive sampling method. Each interview was recorded and transcribed, after which it was analyzed using the qualitative content analysis method.

**Results:**

Three categories were extracted as the challenges and career consequences, and the support they expect to organization of internationally educated nurses: “Language barrier,” “Transition to recover their confidence and increase motivation,” and “Close support from many quarters”. Four categories were extracted as key considerations of support to internationally educated nurses that nursing administrators had recognized: “Clarification of career path”, “Support for insufficient language skills”, “Support for their career reconstruction”, “Comprehensive support”. This research found that nursing administrators had recognized necessity of adequate work‐related support, it almost met the needs of internationally educated nurses. we need to make a clear policy how to develop internationally educated nurses as valuable human resource based on the evidence of further research relate to support practiced at each facility.

**Patient or Public Contribution:**

There was no patient and public involvement since I only conducted interview to nurses in this study.

## 1 INTRODUCTION

The shortage of healthcare human resources is a serious problem worldwide. The shortage of healthcare professionals is a pressing issue because it affects the quality of care provided at hospitals and care facilities (Haddad et al., 2022). A perturbing shortage of approximately 550,000 nurses and caregivers is projected to occur by the end of 2025 (Ministry of Health, Labor and Welfare: MHLW, 2019) in Japan. In a situation where the healthcare labour shortage is becoming increasingly severe, the strategy for managing this serious labour shortage through the entry of foreign workers via the Economic Partnership Agreement (EPA) has attracted attention, and Japanese hospitals also seek to recruit more foreign nurses. Indeed, the number of foreign nurses and care workers recruited based on the EPA system has been increasing (MHLW, 2022); thus, promoting stable employment of internationally educated nurses (IENs) is crucial. In Japan, IEN job descriptions and all employment training depend almost entirely on host hospitals. Even now, more than 10 years after the introduction of EPA in Japan, many issues remain regarding effective training and utilization of IENs. In addition, research is limited on whether they are educated effectively, and what kind of support they expect has not been verified. This indicates that there remains much room for improvement to optimize IEN utilization. Japan accepts nurses from developing countries such as Vietnam, Thailand and the Philippines; thus, we need to comprehend the skill levels of IENs from developing countries and the values attached to these tasks resulting from previous work experiences in their home countries. This study will allow us to better understand the conditions of IENs from developing countries. Discussions about evidence from Western countries that accept more IENs than Japan will result in establishing effective human resource management of IENs. Therefore, this study aimed to explore the hardships, career consequences, and work engagement of IENs and the effective approach to providing career support for IENs by nursing administrators (NAs) to contribute to the intention to continue working. A descriptive interview study with IENs who work in Japanese hospitals and their NAs was conducted, focusing on the following research questions:

1. How do NAs describe their perceptions of fostering IENs?
2. Are NAs who have successfully managed IENs aware of the key requirements for useful support?
3. How do IENs perceive the difficulties of working in a foreign country?
4. How would you describe your career as a nurse in a foreign country?
5. What kind of support do IENs want to develop their career?

## 2 BACKGROUND

Cross‐border migration of nurses has become a global phenomenon, and nurses with global careers have been increasing. IENs are well accepted in Organization for Economic Cooperation and Development (OECD) countries; in 2019, the proportion of foreign‐educated nurses was 26.6% in New Zealand, 25.9% in Switzerland, 18.1% in Australia, 15.4% in the UK and 8.4% in Canada (OECD, 2021). These rates have been increasing in all countries. A study reported that these nurses expect better employment opportunities and higher salaries, learn a new language, land further educational opportunities, seek opportunities for more autonomy as nurses or work as part of a respected team of healthcare providers (Moyce et al., 2016). However, literature reviews on the challenges of IENs mention language and communication barrier difficulties in orienting to the new environment, underutilization of skills, discrimination and racism as some of the factors faced by IENs in general (Pung & Goh, 2017). Class, sex, race and ethnicity‐based disadvantages in hierarchical labour markets, as well as prejudice, verbal harassment and sexual harassment from customers, have been reported (Schilgen et al., 2019). In a review in the USA, five barriers were reported as challenges in the IENs' transition‐to‐practice experience, which included prejudices related to educational readiness, communication and language, cultural differences, differences in nursing practice and legal issues (Ghazal et al., 2020). Only 573 IENs under the EPA system have passed the national licence for nurses in Japan over the past 10 years, which is approximately 5% of all Japanese nurses. A literature review (Ohya, 2018) reported some of the challenges of IENs in Japan, such as language barriers, low National Board Examination (NBE) pass rates, and cultural differences. Regarding job satisfaction, the majority of participants were somewhat satisfied with their jobs (Nugraha & Ohara‐Hirano, 2016). On the other hand, it has also been revealed that they have lower levels of job satisfaction (Ikeda, 2017). In addition, many IENs still have anxiety about their work and communication in Japanese even after passing the NBE (Ito, 2019). This finding means that more than 10 years after the introduction of EPA in Japan, many issues remain regarding the effective training and employment of IENs and that further improvements are needed to optimize IEN employment. Bruyneel et al. (2013) mentioned that the human resource management of IENs from developing countries should pay more attention to professional socialization and continuing education and training. In addition, IENs need not only hard skills that are credentialed by university degree qualifications but also enhanced soft skills, such as communication and customer care skills in host countries (Cooke & Bartram, 2015). Japan mainly accepts nurses from developing countries such as Vietnam, Thailand and Philippines; thus, we need to comprehend the skill levels of IENs from developing countries and the values attached to these tasks resulting from previous work experiences in their home countries. Goto (2018) also pointed out that for educational support of IENs, it is urgent to provide a basic framework for the education system at the host facility, prepare a reference book for national examinations dedicated to IENs, and prepare a guideline that considers individuality. This study will allow us to better understand the conditions of IENs from developing countries. Also, discussions about evidence from Western countries that accept more IENs than Japan will result in providing effective support to IENs.

## 3 OBJECTIVE

To explore, from the perspective of IENs, their hardships, career consequences and work engagement, and the effective approach to career support for IENs from the perspective of NAs.

## 4 METHODS

### 4.1 Design

A qualitative descriptive study was performed. A qualitative descriptive design is particularly relevant where information is required directly from those experiencing the phenomenon under investigation (Bradshaw et al., 2017). This study was reported using Consolidated Criteria for Reporting Qualitative Research (COREQ) guidelines, a 32‐item checklist (Tong et al., 2007).

### 4.2 Sampling and recruitment

This study was conducted in Japan in 2020. IENs and NAs were selected through purposive sampling. First, we searched hospitals that have been accepting IENs by referring to information such as the website of Japan Hospital Associations or each facility (see the organization report) or checked activity reports in journals of nursing. Then, we contacted the director of the selected hospital by email or telephone to explain the research purpose and gain verbal consent. To obtain a richer narrative, we selected “NAs who have experience mainly in the development of human resources for IENs and their utilization in clinical settings” and “IEN who is active as a registered nurse.” Prior to the interview, I sent the participant information sheet that explain the research purpose to each interviewee and gained verbal consent by telephone. On the day of the interview, each interviewee signed a consent form prior to commencing the interview.

### 4.3 Data collection

Semi‐structured interviews (SSIs) were conducted to collect data. SSIs can be a productive method of collecting open‐ended data from participants (DeJonckheere & Vaughn, 2019). In addition, SSIs ascertain participants' perspectives regarding an experience pertaining to the research topic (McIntosh & Morse, 2015). The interviews were conducted by the main author, who had obtained the skill to listen and respond to participants' answers or responses during an SSI. To build a strong, confidential relationship, e‐mails were exchanged with the participants before the interview. All interviews were conducted virtually or by face to face in a private room at each facility. Each interview took approximately 30–60 min. All interviews were audio‐recorded to maintain an audit trail that could be used to trace each step of the analysis back to the original study protocol. The interview was finished when data saturation was reached.

### 4.4 Interview guideline

The interview guide was developed by the researcher with reference to the literature. The interview guide for the NAs consisted of 3 different topics. The first part asked about their considerations regarding the work environment of foreign nurses. These questions included how they assign IENs, who support them, and how they think about the scope of work of IENs. The second part focused on career support for IENs. They were asked about the presence of an in‐facility education system and the challenges faced while training IENs. The third part asked administrators to describe the key factors of effective human resource management of IENs in the clinical setting.

The interview guide to IENs consists of 2 parts. The questions in the first part focused on the hardships of IENs. These questions included whether they find it difficult to work in Japan and whether they have received useful support. The second part focused on their career consciousness. They were asked to express their thoughts on their career awareness as nurses and the support they need.

### 4.5 Data analysis

This study applied qualitative content analysis, which is usually used to analyse a set of texts, such as interview transcripts. This includes categorizing verbal or behavioural data to classify and summarize data (Krippendorff, 2018). As part of the categorization process, the researcher read all verbatim accounts and marked the sections in IENs' verbatim accounts that discussed the difficulties they experienced, the support they received from the organization, and the support they expected in the future. The verbatim accounts of NAs highlighted the support they provided to IENs and the organization's policies. The sections that discussed each of these issues were marked. Other passages relevant to the overall purpose of this study were also marked. These passages were thereafter coded, and these codes were classified according to the similarity in the meaning of the passages and divided into subcategories. These subcategories were classified in terms of broader concepts and assigned a theme. To ensure that the themes represented the interview data accurately, the categories were compared with the transcribed interview data. QSRNVivo was used to code and categorize the data. In the results, the participants' responses to subcategories were calculated based on their numbers using the following terms: “few” for 1–2, “some” for 3, “half” for 4, “most/majority” for 5–7 and “all” for 8 or 9. Narratives reflecting the beliefs of many participants were selected and quoted to provide examples of how the categories and subcategories were derived. All quotes were translated from Japanese into English.

### 4.6 Ethical considerations

Ethical approval was approved by the ethics committee of the related university. Institutional permission was obtained from the hospital where the study was conducted. The participants were explained the purpose of the research and the protection of their rights. They were further asked for their cooperation and provided with a copy of the participant information sheet.

### 4.7 Rigour

This study also used the 4 criteria developed by Lincoln and Guba (1985) to achieve trustworthiness and rigour. These included the following: (1) ensuring credibility by applying purposive sampling and specific, criteria‐based sampling for information to ensure that participants are knowledgeable. Three participants reviewed the result and confirm the accuracy of the themes and subthemes in relation to their personal experiences; (2) ensuring dependability by receiving feedback from an expert senior investigator; (3) ensuring transferability by choosing participants in the same way (as IENs or NAs) and each required to meet strict inclusion criteria and (4) ensuring confirmability by audio‐recording all interviews and maintaining an audit trail that could be used to trace each step of the analysis.

## 5 RESULTS

### 5.1 Participants

The participants were 8 IENs and 9 NAs. Two IENs were in their 20s and six were in their 30s. They were from Indonesia (3), Vietnam (3) and the Philippines (2). Six IENs attended nursing or junior colleges, and four attended nursing universities. Two nurses had >4 years of experience, one had >3 years, and five had 2 years. The average interview time was 29.3 (range, 25–35) min (Table 1). NAs comprised two nursing education managers, one vice president, one deputy hospital director, one director of nursing, one deputy director of nursing and three nurse managers. The average interview time was 31.6 (range, 25–40) min (Table 2).

**TABLE 1:** Characteristics of the participants (IENs).

No	Age	Home country	Academic background	Nurse experience in home county	Length of Interview(min)
N1	20s	Vietnam	BSN	2 years	25
N2	20s	Vietnam	DNS	2 years	35
N3	30s	Vietnam	BSN	2 years	35
N4	30s	Indonesia	DNS	3 years 6 months	30
N5	30s	Indonesia	DNS	2 years	25
N6	30s	Indonesia	DNS	2 years	30
N7	30s	Philippines	BSN	4 years 10 months	30
N8	30s	Philippines	BSN	4 years	25

Abbreviations: BSN, Bachelor of Science in Nursing; DN, Diploma in Nursing Science.

**TABLE 2:** Characteristics of the participants (Administrators).

No	Position	Length of interview(min)
A1	Nursing Education manager	35
A2	Nursing Education manager	40
A3	Vice‐president	30
A4	Vice‐president Director of Nursing	30
A5	Director of Nursing	25
A6	Deputy Director of Nursing	25
A7	Nurse Manager	30
A8	Nurse Manager	40
A9	Nurse Manager	30

#### 5.1.1 Interview with IENs

Eight categories were extracted as hardships experienced by IENs, effect on their careers, and support they sought. Then, these categories were categorized into 3 themes: language barrier, transition to recover their confidence and increase motivation, and close support from many quarters (Table 3).

**TABLE 3:** IENs' difficulties, and support they need to.

Theme	Category	Subcategory	Rating
Language barriers	Communication difficulties with patients	I do not understand what the patient says or the dialect	5
		I cannot hear what the elderly are saying	5
		I am depressed as I am unable to answer patients' queries	2
	Difficult medical terms and kanji	I cannot read technical terms or kanji	3
		I cannot follow the conversations of doctors and nurses because they talk too fast	3
		Struggling to improve Japanese language skills, pronunciation, and grammar complex	3
Transition to recover their confidence and increase motivation	Loss of confidence as a nurse	Patients do not recognize me as a nurse. I feel inferior to new nurses in Japan	3
		Impatient for not being able to perform a full‐fledged job as a nurse even after passing the national exam	2
		I do not feel confident working in Japan	5
	Expansion of the role as a nurse	The more a person can do, the more they feel that they are useful	3
		The more you can do, the more interesting your work becomes	3
		I am now able to take care of patients on my own, and I am hopeful about working in Japan	4
	Willingness to learn new things	Target a professional qualification	2
		Allowing time to realize your goals	2
		Want the challenge of working on a busier ward	4
		I want to learn new nursing techniques and treatment procedures	4
Close support from many quarters	Mentors in the field	People who are quick to tell you when you face a problem in the ward	3
		A work environment where you can ask for and receive support immediately	3
		Support for patient communication	3
	Peer support	Advice on solving work and personal problems	5
		Emotional support by conversing in native language	3
		Guidance on nursing tasks from senior FENs	3
	Satisfaction with treatment of employees	Living support from the host organizations	3
		Sufficient salary	4

##### Language barriers

All IENs said they struggled with a “language barrier,” that is, lacking sufficient Japanese language proficiency to play the role of a nurse in a clinical setting. Two subcategories related to this theme were identified.

(a) Communication difficulties with patients.

Most IENs (N1–N4 and N7) stated that they were unable to understand what the patient says or the dialect and language of older patients. They felt depressed because they could not understand the patient's complaints, and they could not provide care as a nurse; as a result, they felt that they were considered bad nurses.

“Communication is…it's still hard. I can't understand what the patients are saying. Dialects and such… it's difficult to understand what they mean if you're not used to it.” [N2]

“Sometimes it's difficult to hear what the patient is saying. Huh? … What did you just say? Many of the patients are old, so it's difficult to understand what they are saying. Also, there is a dialect, so there are quite a few times when I don't understand.” [N1]

(b) Difficult medical terms and Chinese characters/kanji.

The following subcategories were identified in this category. Some IENs (N2–N4) said that they were nervous when reporting to doctors and that they could not keep pace with the speed at which nurses conversed with each other. Many difficult kanji are used for medical purposes, such as diseases and internal organs. Even though they had acquired the necessary level of Japanese and passed the NBE, they could not keep up with the jargon used in clinical settings and the fast conversations of nurses and doctors.

“The technical terms and Chinese characters used in the general wards are difficult to comprehend, and the nurses speak so fast that the reports are inaudible to me. It makes me very nervous.” [N3]

“I thought I could work as a routine nurse after passing the national exam, but it's difficult to speak Japanese. Even now, there are times that I can understand what the patient is saying, and sometimes, I can't.” “What's that? I've never heard of that at all.” [N4]

##### Transition to recover their confidence and increase motivation

In the process of integration into Japanese workplaces, their inability to provide nursing care lowered their self‐confidence and caused insecurity about their career. However, as their roles as nurses expanded, they increased their motivation to work by wanting to learn more when they were given new tasks.

(a) Loss of confidence as a nurse.

Some IENs (N1, N2 and N4) believed that they were not recognized as “nurses” by patients and that they could not perform their jobs as full‐fledged nurses, which made them feel hopeless about continuing to work in Japan.

“I don't feel a sense of satisfaction that I'm useful. There are different ways of doing things, and I don't know a lot of things.” [N1]

“They think, ‘this nurse, no good’… It's so hard. I can't perform my job any better than a new nurse in Japan … I feel like I can't be useful. I feel insecure, and I don't feel satisfied with my job.” [N4]

(b) Expansion of their role as a nurse.

Some IENs (N1, N2 and N4) mentioned that they experienced an initial period of a lack of confidence as a nurse, which decreased their self‐esteem. Thereafter, they noticed that the longer they continued working and as more responsibilities were given to them, their level of confidence increased. Hence, they now have a sense of fulfilment in their work and hope to work in Japan.

“The more I can do, the more I feel like I'm useful. It also makes work more interesting.” [N1]

“I want to work in Japan for another 2–3 years. I'm able to perform most of the tasks now, so… I think I'll be okay.” [N2]

(c) Willingness to learn new things.

Half of the IENs (N2 and N4–N6) said that they had been at their current place of work for more than 2 years and were now ready to consider new challenges to improve their knowledge and skills as nurses.

“I have been working in my current ward for 2 years and have become capable of performing my duties, so I would like to try another general ward. I would like to work in Japan a little longer.” [N2]

“I want to become a professional nurse, and I'm studying for that.” [N5]

##### Close support from many quarters

Three subcategories related to close support from multiple sources were identified. Half of the IENs (N1–N4) answered that support from other nurses (mentors) and senior IENs was important. In addition, they felt that the support and salary from the organization were sufficient.

(a) Mentors in the field.

Some IENs (N1, N3 and N4) wanted to have a mentor in the clinical setting and an environment in which they could easily receive support from other nurses.

“The head of the ward is very kind and guides me through a lot of things … That's really good. Other nurses are busy, and it's difficult to ask questions and stuff. The chief cares about me.” [N3]

(b) Peer support.

Most IENs (N1–N4 and N7) considered the advice and interaction from senior IENs as emotionally support. Some (N2–N4) participants said that communication in their native language relieved their stress and provided a sense of security.

“My senior took me shopping and stuff … taught me a lot.” [N2]

“They listen to my concerns about work and support me emotionally.” [N3]

(c) Satisfaction with the treatment of employees.

Some participants (N2–N4) mentioned satisfaction with living support from the host organizations and salary.

“The hospital provides a lot of support, for 3 years after coming to Japan, the hospital provides a dormitory.” [N3]

“I'm still not satisfied with my job … but I'm getting enough money.” [N4]

#### 5.1.2 Interview with NAs

Four themes were extracted as considerations regarding IENs: “clarification of career path,” “support for insufficient language skills,” “support for their career reconstruction,” and “comprehensive support.” (Table 4).

**TABLE 4:** Administrator's' view

Theme	Category	Subcategory	Rating
Clarification of career path	Use of Newcomer Education program	Training in the same educational program as new graduate nurses	7
		Confirmation of skills using the same nursing skills checklist as for new graduate nurses	
	Setting of nursing practice skill achievement goals	The clinical ladder established by Japan Nursing Association to determine the nursing practice skills of FENs	4
		Establishing criteria for allowing a nurse to work on night shifts	
		The need to tool to measure proficiency for FENs	
Support for improving language skills	Japanese education in line with clinical setting	Ongoing support to improve language skills	4
		Assist in answering phone calls and reporting to physicians	
		Recognize that it takes more time to mature as a nurse than as a Japanese nurse	
	Support in the clinical setting	Responding to patient complaints about FENSs and providing emotional follow‐up to FENs	2
Support for their career reconstruction	Provide opportunities for growth	Give them responsibility and help them choose a career path as a nurse.	4
		Gradually expand the role of work by transferring to a ward that requires more skill	
		Workplace transfers as they grow	
		Provide opportunities to learn new technologies	
	Utilization of existing abilities	Utilize my nursing experience acquired in my home country in the workplace	3
		Provide opportunities to help them play an active role in managing foreign patients. Utilize them in the right place at the right time	
	Clarification of individual goals	Identify their hopes for the future and work towards achieving them	5
		Nurturing EPA nurses as human resources who can contribute to medical and nursing care in their home countries	
		Make EPA nurses set their own goals	
Comprehensive support	Support for a stable life	Support to achieve stability in life and mental health	5
		The presence of people who support you in general outside of work	
		Consideration for family in the home country and granting long holidays	
	Organizational culture that accepts workforce diversity	Dispel prejudice against foreigners in the organization	5
		Staff's common understanding of the acceptance of foreign nurses	

##### Clarification of career path

Most NAs stated they train IENs in the same program as new nurses in Japan. The NAs expected IENs to learn the Japanese nursing system and care, and they provided IENs with an education that followed Japan's newcomer education, despite IENs having work experience as a nurse in their home country. In addition, half of the NAs (A4 and A6–A9) had set a clear skill level and attainment for IENs, although some of the NAs struggled with where to set it. As IENs have various backgrounds and organizations require nurses to have different abilities and roles, the goals of IENs as nurses depended on the host hospital.

(a) Use of newcomer education program.

Most participants (A1 and A3–A9) stated that IENs participate in an education program for new graduate nurses, and they evaluate nursing skill acquisition using the checklist of new graduate nurses.

“They (IENs) participate in the education program for graduate new Japanese nurses. I think it's less discriminatory or lonely for them to be with Japanese people, and I want them to develop a sense of camaraderie.” [A1]

“They already have nursing experience in the Philippines, but we are educating them as one of our newcomers. This also means that they make friends with new graduate nurses in Japan. We used the same checklist to confirm nursing skills as new graduate nurses.” [A4]

(b) Setting of nursing practice skill achievement goals.

The goals for the attainment of nursing practice skills for IENs differed based on individual hospital policies and were set by the clinical ladder of the Japan Nursing Association (A1) or the hospital's clinical ladder (A2, A4–A7).

“We decided to refer to the clinical ladder recommended by the Japan Nurses Association and use Level II of that ladder as the goal for the level of achievement for IENs.” [A1]

“We develop them by having them participate in the same education as other nurses, depending on their level of development, such as leadership training.” [A5]

##### Support for insufficient language skills

Some NAs (A6–A8) considered that IENs have difficulty communicating with patients in the clinical setting and sharing information and communicating with doctors and nurses. Thus, they provided IENs with language education in addition to the new nursing program to improve Japanese language skills and considered dealing with troubles with patients.

(a) Japanese education in line with the clinical setting.

Some NAs (A6–A8) provided additional education to the new nursing program to improve the Japanese language skills of IENs.

“In addition to the new nurse program, we provide education on communication, nursing records, and other topics concerning the wards. We also provide additional education in the nursing management department as deemed necessary in consultation with the individual.” [A6]

(b) Addressing issues associated with the lack of language skills.

Few participants (A1 and A6) noted the importance of responding to communication problems between patients and IENs and the subsequent emotional follow‐up with IEMs.

“Occasionally, patients complained about them, saying, ‘You're not good enough,’ or ‘Go get a Japanese nurse.’ I think this is unavoidable. However, I believe that it is important to follow‐up mentally with IENs afterwards.” [A3]

##### Support for their career reconstruction

Three categories of this theme were identified. NAs considered working in busy wards according to the growth of nursing practice skills rather than fixing IENs to work in less urgent wards with fewer nursing procedures. In addition, NAs perceived considered the mother language and English proficiency as the strengths of IENs.

(a) Provide opportunities for growth.

Half of the NAs (A1, A2, A4 and A5) mentioned the need to consider their place of work according to their ability to practice nursing.

“We are considering assigning them on respiratory and dialysis wards. These wards do not have a lot of admissions and discharges, and the work is not too busy… Once they get used to the work, I'll consider transferring them to wards with a bit more urgency and severity of patient conditions.” [A4]

(b) Utilization of existing abilities.

Some NAs (A2, A4 and A5) considered their mother language, English proficiency and clinical experience in their home countries as the strengths of IENs.

“We have a lot of foreign patients in our hospital. IENs can communicate with them in English. I think the key is utilizing their strengths and assigned them in the right places.” [A4]

(c) Clarification of individual goals.

Most NAs (A1, A2, A4, A5, A7 and A9) stated that their policy was to help IENs set personal goals.

“We have confirmed what kind of career he wants to pursue, but he said that he will work hard at his current job for now, and we want to support that.” [A5]

“We should support them to keep their motivation to retain their work in Japan. We need to think about their careers, taking into account their future aspirations and whether they want to continue working in Japan or return to their home country.” [A2]

##### Comprehensive support

All NAs stated that efforts and organizational culture to accept IENs is important for the development of the IEN workforce. Two subcategories related to comprehensive support by the organization were identified.

(a) Support for a stable life.

Some NAs stated (A1–A3) that the people who can solve their concerns are crucial for IENs to maintain a stable life.

“Of course, the work aspect is important, but support regarding the life aspect is also essential. We need to have a person in charge who can help them solve their problems in life. I think it's important to support them so that they can stabilize their life, their feelings, or their mentality.” [A2]

(b) Organizational culture that accepts workforce diversity.

Most NAs (A1–A5 and A9) stated that a common understanding within the organization that does not perceive working with foreigners negatively is beneficial for the development of IENs.

“I think the smooth transition of IENs into the hospital can be attributed to the entire staff's understanding of them. It is through this trial and error that we are able to educate and utilize them today.” [A2]

## 6 DISCUSSION

The main finding of this study is about language barriers. Barriers to Japanese proficiency were realized as a major problem for both IENs and NAs. Language and communication barriers have been reported as issues in many studies targeting IENs. A recent systematic review of research on IENs in Japan reported language and communication barriers as issues IENs are facing (Abuliezi et al., 2021). However, these findings mainly focus on Japanese language learning during the period until passing the NBE is being considered; however, no study has reported support in the clinical setting after passing the NBE. Japan Language Proficiency Test (JLPT) N1 is a prerequisite for licensed foreign nurses to take the Japanese National Examination for Nurses (MHLW, 2020). This study found that IENs still experience difficulty in reading kanji and understanding dialects in the clinical setting even though they have JLPT N1. In an acute care ward, communication skills in Japanese are indispensable for clinical judgement, and prompt judgement and flexible coping are required in the acute clinical setting where verbal instructions are exchanged. In recovery wards, health guidance for patients is a major nursing task. Language and communication barrier is a main stressor that impedes functioning, team collaboration and positive nurse–client relationship (Schilgen et al., 2019), and it can be a major obstacle to patients and healthcare professionals in the clinical environment. Even Japanese medical students are required to have a Japanese Language Examination pre‐level 1 or level 1, to read and understand nonstandard Chinese characters/kanji that are often used in medical terms. Considering these conditions, the author recommends developing human resources equivalent to the JLPT N1 level to expect IENs to succeed in the medical field. In other developed countries, IENs are required to take an educational program at an educational institution such as a university, which allows trainees to take a program tailored to their abilities without relying on the receiving institution for their education. The USA established the Commission on Graduates of Foreign Nursing Schools in 1977 to ensure foreign nurses' technical and cultural competence before employment in US healthcare institutions (Xu et al., 1999). In the UK, the Nursing and Midwifery Council had set strict requirements for foreigners to obtain a nurse licence to ensure the quality of their nurses: acquisition level by International English Examination System, nursing skill test by objective structured clinical tests, and computer‐based tests to measure nursing knowledge (The Nursing and Midwifery Council, 2021). Therefore, to ensure the quality of IENs and achieve success in clinical practice, we believe that the development of standards for judging Japanese language proficiency and the development of continuing education systems are priorities. In addition, field managers must create an environment in which the quality of medical care and nursing can be maintained while compensating for the lack of Japanese language skills of IENs. In addition, IENs have different specialities depending on the department to which they are assigned, and they are required to acquire the necessary technical terms and Japanese expressions. Considering that teaching materials in such cases are highly specialized and individualized, it is challenging to create them uniformly, as in the preparation for the national examination (Ito, 2019). However, having a relationship in practice where IENs can feel that their abilities are being utilized and developing and maintaining educational support that motivates them should be effective.

Second, human resources development must support and enable IENS to maintain their self‐esteem and growth as nurses. In this study, most IENs participated in the education of new graduate Japanese nurses, despite their experience as nurses in their home countries. Ito (2019) reported that the challenges encountered by IENs in the process of adjusting to the workplace differ from those of new nurses in Japan, and they can be attributed to differences in nursing views and nursing skills learned in their home countries. Improving foreign nurses' knowledge of professional and social practices that may be different from those in their home country may help ease the transition to another culture (Sherman & Eggen, 2008). There are essential differences in the basic education of nurses in different countries with respect to the quality, scope, and duration of education. Education programs for new nurses are comprehensive and include nursing skills, communication and Japan medical system. Thus, participation in new nurse education may lead to the acquisition of knowledge by IENs. IENs were motivated by the expansion of the range of nursing tasks that they could perform, and the transfer to a new department led to increased motivation and confidence. Bland and Woolbridge (2011) reported that new skills learned and growth opportunities motivate them both professionally and personally. Furthermore, the utilization of expertise in their home country is effective human resources management. The source country and professional experience influence education outcomes in host countries (Covell & Rolle Sands, 2021). Therefore, to make IENs an effective human resource, the kind of growth opportunities that they desired individually must be taken into account, considering their cultural backgrounds, nursing views and experience in their home countries. Western countries offer educational support to IENs, such as the transition‐to‐practice in the USA (Ghazal et al., 2020) and bridging programs in Canada (Covell & Rolle Sands, 2021). Covell and Rolle Sands (2021) reported that bridging programs help IENs address gaps in their cultural, practical, and theoretical knowledge. Thus, these workplace integration programs are useful in Japan to assist IENs.

Third, this study noted “comprehensive support.” IENs hoped to support mentors and peer support from senior IENs for leading a stable life in Japan. Specifically, trust among nurses and a workplace atmosphere in which “anything that is not understood is immediately asked (can be asked)” are meaningful for IENs. Administrators stated the importance of organizational support for a stable life and the formation of an organizational culture that accepts foreigners, and their support must fully meet the needs of IENs. Each IEN has a different cultural and religious background, nursing experience, Japanese language ability and communication skills (Otani, 2018). Communication and language differences, the thought of being an outsider, and differences in nursing practice led to a feeling of alienation (Newton et al., 2012). Migrant nurses of different origins perceive their status as migrants as a sense of community by sharing the same destiny; this appears as an important resource for migrant nurses (Ghazal et al., 2020). Providing education, managerial support and mentorship fosters IENs' workplace integration (Covell & Rolle Sands, 2021). Thus, creating a work environment where IENs can work with peace and mental support is important.

### 6.1 Implications of this study

Based on the results, we propose the following recommendations for the effective utilization of IENs as human resources and for IEN's career development in foreign countries. It is also effective to develop and maintain educational support that motivates IENs by building practical relationships where they can feel that their abilities are being utilized.

- Continued support to improve Japanese language skills.
- Creation of guidelines with goals considering the career development of IENs.
- Clarify the individual goals of IENs.

### 6.2 Limitations and recommendations

This study had a small sample size, had self‐selection bias due to participation by volunteers, and had limited hospital type, scale and area. Therefore, it was not possible to fully extract the diversity of this theme as a qualitative study. However, we were able to show specific problems and direction of efforts in the clinical setting from the narratives of IENs and NAs.

## 7 CONCLUSIONS

This study found that IENs faced language barriers and lost their confidence as a nurse. In addition, IENs were satisfied with the support by organization. Meanwhile, key considerations for IENs that NAs had recognized were insufficient language skills, need to reconstruct their career as nurses and workforce diversity. Thus, developing a workplace integration program in Japan based on the evidence of support practised at each facility is necessary.

## AUTHOR CONTRIBUTIONS

Chiharu Miyata designed the study, conducted interviews, analysed data, and drafted and finalized the manuscript.

## FUNDING INFORMATION

This work was supported by an education and research grant funded by the Mie University, Japan.

## CONFLICT OF INTEREST STATEMENT

The authors declare there are no conflict of interests.

## Data Availability

Data sharing is not applicable to this article as no new data were created or analyzed in this study.
